# Six Weeks of Low-Load Blood Flow Restricted and High-Load Resistance Exercise Training Produce Similar Increases in Cumulative Myofibrillar Protein Synthesis and Ribosomal Biogenesis in Healthy Males

**DOI:** 10.3389/fphys.2019.00649

**Published:** 2019-05-29

**Authors:** Peter Sieljacks, Jakob Wang, Thomas Groennebaek, Emil Rindom, Jesper Emil Jakobsgaard, Jon Herskind, Anders Gravholt, Andreas B. Møller, Robert V. Musci, Frank V. de Paoli, Karyn L. Hamilton, Benjamin F. Miller, Kristian Vissing

**Affiliations:** ^1^ Section for Sports Science, Department of Public Health, Aarhus University, Aarhus, Denmark; ^2^ Department of Biomedicine, Aarhus University, Aarhus, Denmark; ^3^ Steno Diabetes Center Aarhus, Aarhus University Hospital, Aarhus, Denmark; ^4^ Department of Health and Exercise Science, Colorado State University, Fort Collins, CO, United States; ^5^ Aging and Metabolism Research Program, Oklahoma Medical Research Foundation, Oklahoma City, OK, United States

**Keywords:** ischemic resistance training, deuterium oxide, remodeling, myofibrillar protein synthesis, ribosomal biogenesis

## Abstract

**Purpose:** High-load resistance exercise contributes to maintenance of muscle mass, muscle protein quality, and contractile function by stimulation of muscle protein synthesis (MPS), hypertrophy, and strength gains. However, high loading may not be feasible in several clinical populations. Low-load blood flow restricted resistance exercise (BFRRE) may provide an alternative approach. However, the long-term protein synthetic response to BFRRE is unknown and the myocellular adaptations to prolonged BFRRE are not well described.

**Methods:** To investigate this, 34 healthy young subjects were randomized to 6 weeks of low-load BFRRE, HLRE, or non-exercise control (CON). Deuterium oxide (D_2_O) was orally administered throughout the intervention period. Muscle biopsies from m. vastus lateralis were collected before and after the 6-week intervention period to assess long-term myofibrillar MPS and RNA synthesis as well as muscle fiber-type-specific cross-sectional area (CSA), satellite cell content, and myonuclei content. Muscle biopsies were also collected in the immediate hours following single-bout exercise to assess signaling for muscle protein degradation. Isometric and dynamic quadriceps muscle strength was evaluated before and after the intervention.

**Results:** Myofibrillar MPS was higher in BFRRE (1.34%/day, *p* < 0.01) and HLRE (1.12%/day, *p* < 0.05) compared to CON (0.96%/day) with no significant differences between exercise groups. Muscle RNA synthesis was higher in BFRRE (0.65%/day, *p* < 0.001) and HLRE (0.55%/day, *p* < 0.01) compared to CON (0.38%/day) and both training groups increased RNA content, indicating ribosomal biogenesis in response to exercise. BFRRE and HLRE both activated muscle degradation signaling. Muscle strength increased 6–10% in BFRRE (*p* < 0.05) and 13–23% in HLRE (*p* < 0.01). Dynamic muscle strength increased to a greater extent in HLRE (*p* < 0.05). No changes in type I and type II muscle fiber-type-specific CSA, satellite cell content, or myonuclei content were observed.

**Conclusions:** These results demonstrate that BFRRE increases long-term muscle protein turnover, ribosomal biogenesis, and muscle strength to a similar degree as HLRE. These findings emphasize the potential application of low-load BFRRE to stimulate muscle protein turnover and increase muscle function in clinical populations where high loading is untenable.

## Introduction

Disease and advanced ageing can negatively affect muscle mass (Lexell et al., 1988; Mancini et al., 1992; Lindle et al., 1997) and muscle protein quality (Haus et al., 2007; Gouveia et al., 2017), which can contribute to impaired muscle contractile function (Harrington et al., 1997; Lindle et al., 1997; Brocca et al., 2017). Reductions in muscle mass and function can negatively affect mobility (Visser et al., 2005) and all-cause mortality (Metter et al., 2002; Szulc et al., 2010). Muscle mass and protein quality are determined by protein turnover. In this regard, a net positive muscle protein synthesis (MPS) increases muscle mass (Damas et al., 2016), while equivalent increases in MPS and protein degradation may reflect remodeling to maintain protein quality (Miller et al., 2014; Musci et al., 2018). Stimulating protein turnover during ageing and disease may therefore be important for preserving a functional muscle mass.

A single-bout of high-load resistance exercise (HLRE) is known to stimulate acute (~24–72 h) transient increases in net MPS (Phillips et al., 1997; Miller et al., 2005). Recent studies using deuterium oxide (D_2_O) tracer methodology have demonstrated that prolonged HLRE training can stimulate cumulative increases in MPS to produce muscle hypertrophy (Brook et al., 2015; Damas et al., 2016). This impact of HLRE on MPS may partially rely on the translational capacity and activity of ribosomes (West et al., 2016). Accordingly, ribosomal biogenesis is reported to be associated with increased myofibrillar MPS (Brook et al., 2017) and muscle hypertrophy (Stec et al., 2016). In addition, satellite cell-mediated addition of myonuclei to existing muscle fibers has been proposed to contribute to muscle hypertrophy (Petrella et al., 2008), although the notion on the importance of satellite cells for muscle hypertrophy has later been challenged (McCarthy et al., 2011).

Some clinical conditions (e.g., arthritis or recovery from orthopedic surgery) may impede the use of high loading to counteract loss of muscle mass and function. In this regard, it is interesting that low-load resistance exercise regimens have proven effective at stimulating MPS as well as at promoting muscle hypertrophy and strength gains (Burd et al., 2010; Mitchell et al., 2012) in young individuals. On the other hand, low load training entails a high work volume, so it is relevant to consider alternative training regimes.

Exercise with simultaneous blood flow restriction (i.e., low-load blood flow restricted resistance exercise, BFRRE) reduces the volume of low-load exercise needed to stimulate muscle growth and strength (Fahs et al., 2015; Farup et al., 2015). Accordingly, a single bout of BFRRE has been reported to stimulate MPS (Fujita et al., 2007; Fry et al., 2010) and ≤ 6 weeks of BFRRE training has been reported to produce strength gains as well as both whole-muscle and fiber hypertrophy (Nielsen et al., 2012; Farup et al., 2015; Bjornsen et al., 2018a,b). Moreover, studies by Nielsen et al. (2012) and Jakobsgaard et al. (2018) observed increases in satellite cell and myonuclei content with ≤6 weeks of BFRRE training (Nielsen et al., 2012; Jakobsgaard et al., 2018). Interestingly, contrary the preferential type II fiber hypertrophy often seen with HLRE (Aagaard et al., 2001; Campos et al., 2002; Folland and Williams, 2007), BFRRE may direct the stress and hypertrophy more toward type I fibers (Cumming et al., 2014; Bjornsen et al., 2018a).

The myocellular responses to prolonged BFRRE are not well described. Therefore, the primary aim of the current study was to conduct a randomized controlled trial to investigate the accumulated effects of 6 weeks of BFRRE or HLRE on myocellular adaptations relating to accumulated MPS, RNA synthesis, muscle hypertrophy and strength. A secondary aim was to investigate the accumulated training effects on satellite cell and myonuclei content. We hypothesized; (1) that prolonged low-load BFRRE would be equally effective as HLRE in stimulating MPS and ribosomal biogenesis, and; (2) that both training regimens would stimulate increases in muscle fiber CSA, satellite cell content, and muscle strength.

## Materials and Methods

### Subjects

Thirty-four healthy, untrained male subjects were included in the study [mean (95% CI), age 23.7 (22.9, 24.6) years; height 180.0 (178.2, 181.8) cm; weight 79.0 (74.9, 83.1) kg]. Exclusion criteria were; (1) resistance training within 6 months prior to inclusion; (2) participation in moderate/high intensity exercise training (other than resistance training) more than 1 h/week 6 months prior to inclusion; (3) use of prescription medication or intake of dietary supplements potentially affecting muscle metabolism and growth. Written informed consent was obtained from all participants prior to inclusion. The study was approved by the Central Denmark Region Committee on Health Research Ethics (1-10-72-218-16) and registered in the database clinicaltrials.gov (NCT03380663). The study conformed to the standards for human experimental trials outlined in the Declaration of Helsinki. Results on muscle mitochondrial and metabolic adaptations from the study has been previously published (Groennebaek et al., 2018).

### Study Design

**Figure 1:**
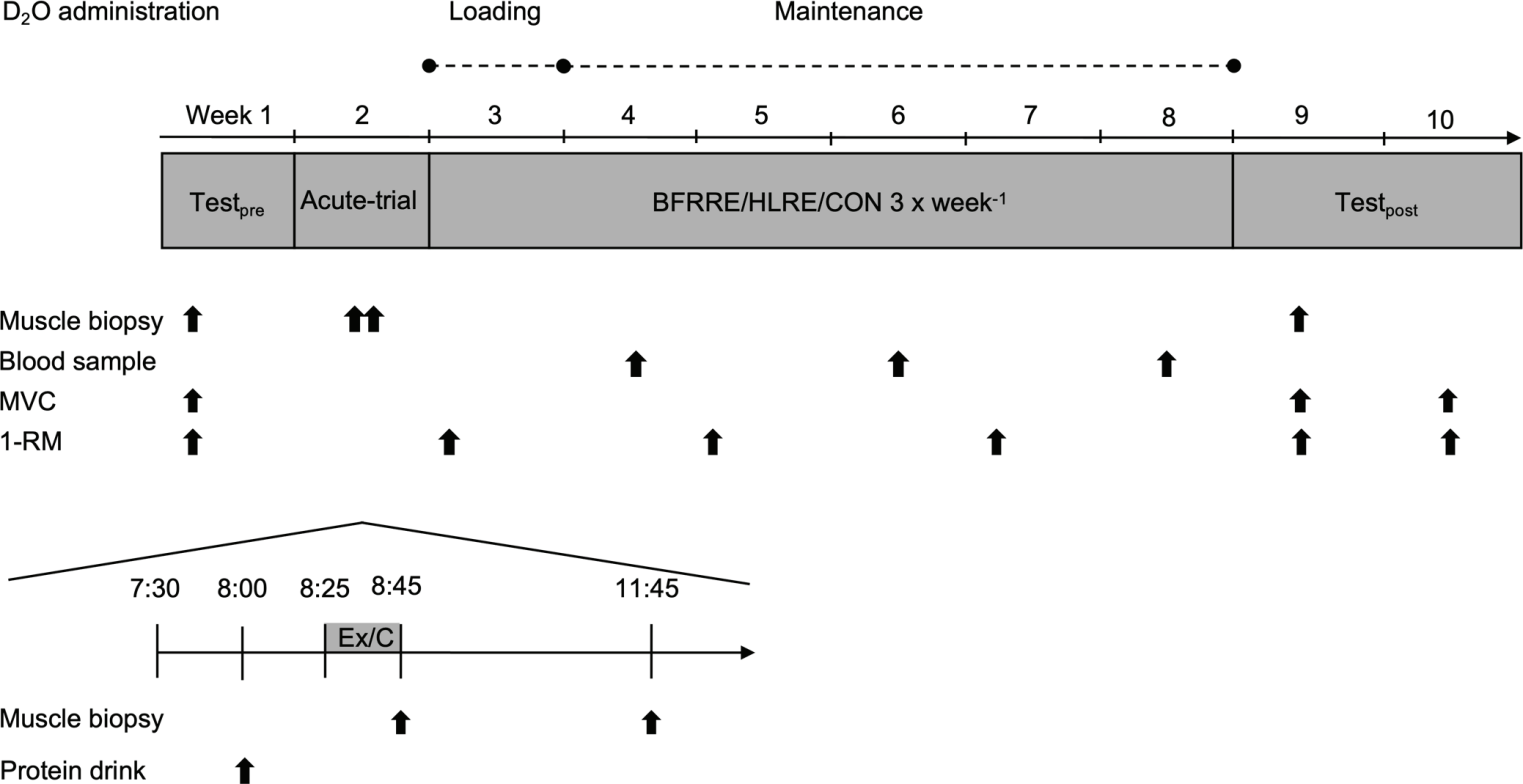
Study overview. Pre-test was conducted 2 weeks prior to the 6-week training period. MVC maximal voluntary contraction; 1-RM one-repetition maximum; Ex, exercise; C, control.

Subjects were randomized to 6 weeks of low-load blood flow restricted resistance exercise (BFRRE, *n* = 12), high-load resistance exercise (HLRE, *n* = 12), or non-exercise control (CON, *n* = 10). Subjects in CON completed all experimental procedures except exercise. The study design comprised of an acute-trial study and a long-term study (Figure 1).

In the acute-trial, subjects arrived at the laboratory after an overnight fast. Subjects rested for 30 min and then consumed a protein drink containing 20 g of whey protein isolate (Whey 100 Extra Pure, Bodylab, Denmark). The protein drink was provided in the acute study to be consistent with common strength training practices. Following consumption of the protein drink, subjects performed a standardized warm-up and a single exercise bout (described in detail later). Muscle biopsies were harvested immediately (0 h) and 3 h post-exercise to assess targets related to autophagy.

For the long-term study, muscle strength tests and collection of skeletal muscle biopsies were performed before and 4 days after completion of the 6-week training period. The BFRRE and HLRE subjects repeated the muscle strength tests 14 days after completing the training intervention to assess possible delayed muscle strength adaptations following BFRRE training (Nielsen et al., 2017). To assess long-term protein synthesis rates, D_2_O was orally administered throughout the training period. Collection of muscle biopsies and tests of muscle strength were performed in the early morning after overnight fasting. Subjects were instructed to maintain their habitual level of physical activity during the intervention period and to refrain from strenuous physical activity and alcohol for 3 days prior to all tests.

### Deuterium Oxide Administration

Oral administration of D_2_O (99.8%, Sigma Aldrich, St. Louis, Missouri, USA) was based on previous studies (Scalzo et al., 2014; Miller et al., 2015; Konopka et al., 2017) and has been described in detail previously (Groennebaek et al., 2018). The first week of the 6-week intervention included an initial loading period with subjects receiving 2 × 1 ml/kg bodyweight on the first day and 1 × 1 ml/kg bodyweight on the following 6 days. For the remaining 5 weeks, the subjects received 1 × 1 ml/kg bodyweight every second day. Plasma D_2_O enrichment was assessed at the end of weeks 2, 4, and 6.

### Blood Flow Restriction

Standardization of blood flow restriction for subjects in the BFRRE group was achieved by prescribing cuff pressures relative to individually determined arterial occlusion pressures (AOP) as previously described (Sieljacks et al., 2017). In short, AOP was determined in a supine position by incrementally inflating a 14-cm pneumatic cuff (Delfi Medical, Vancouver, Canada) using a digital tourniquet (A.T.S 2200TS, Zimmer Surgical Inc. Ohio, USA). Pressure was increased until auscultatory pulse was no longer detectable in the posterior tibial artery using Doppler ultrasound (Dopplex-D900, Huntleigh Healthcare Ltd., UK). AOP was determined at pre and was reassessed at training bout 9. Cuff pressure was set to 50% of AOP during BFRRE. Mean [95% CI] cuff pressure was 79 [74, 84] mmHg in training bout 1–8 and 78 [76, 82] mmHg in bout 9–18.

### Training Protocols

A detailed description of the training intervention has previously been published (Groennebaek et al., 2018). Exercise was practiced in accordance with recommended principles (i.e., BFRRE; low load, many repetitions, short inter-set recovery and HLRE; high load, few repetitions, long inter-set recovery) (ACSM, 2009; Scott et al., 2015). In short, subjects in the two training groups underwent supervised BFRRE or HLRE training 3×/week for 6 weeks. Each training session consisted of a standardized warm-up and a main training bout. For subjects allocated to BFRRE, training bouts consisted of four sets of knee-extension exercise during continuous blood flow restriction (50% of AOP). The training load was set to 30% of 1-RM (repetition maximum) and inter-set recovery was 30 s. For subjects allocated to HLRE, training bouts consisted of knee-extension exercise in four sets of 10–12 repetitions with 3 min inter-set recovery. The training load was set to 70% of 1-RM. For both training groups, the training load was re-adjusted to a corresponding 3-RM test, two times during the 6-week intervention period (weeks 3 and 5). Furthermore, the training load for HLRE was increased if 12 repetitions could be completed for all 4 training sets and decreased if subjects were unable to perform 10 repetitions in the first training set.

### Unilateral Maximal Isometric Knee Extensor Muscle Strength

Following a 5-min warm-up consisting of low intensity (~100 W) ergometer biking (Monark Ergomedic 818E, Monark, Varberg, Sweden), the subjects were seated on an isokinetic dynamometer (Humac Norm, CSMI, Stoughton, Massachusetts, USA) with restraining straps put on the torso and hips to avoid accessory movements. Subjects were positioned with 90° hip flexion and the rotational axes of the knee and the dynamometer lever arm aligned. The subjects’ dominant leg was attached to the dynamometer arm ~3 cm proximal to the medial malleolus. Maximal voluntary contraction (MVC) was measured at 70° knee-flexion (0° = full extension). Subjects were instructed to initiate the contraction as “fast and forcefully as possible” and to avoid any countermovement. Each contraction lasted ~3 to 4 s and subjects were given verbal encouragement and visual feedback during the trials. A minimum of four trials were given and trials were separated by 1 min recovery time. Additional trials were provided to subjects that continued to improve. Torque recordings were sampled at 1,500 Hz and analyzed offline using a custom-made software (Labview 2011, National Instruments Corporation, Austin, Texas, USA). All trials were visually inspected and trials displaying countermovement at the onset of contraction, were excluded from the analysis. MVC was defined as the peak torque recording from the included trials. Test of MVC was conducted at pre, at 4, and 14 days post-exercise.

### Dynamic Maximal Knee-Extensor Muscle Strength (3-RM)

Bilateral knee-extensor 3-RM was assessed in a knee-extension machine (Technogym Selection Line Leg Extension, Technogym SpA, Cesena, Italy) on six occasions; at pre-testing (used for familiarization), immediately before the first training bout in week 1 (used as pre-value), at the first training bout in training weeks 3 and 5 (used for adjustment of training load), at 4 days post-exercise, and at 14 days post-exercise. Warm-up consisted of 5 min of low-intensity (~100 W) ergometer biking (Monark Ergomedic 818E, Monark, Varberg, Sweden) and two warm-up sets of five repetitions with loads corresponding to 50 and 70% of estimated 1-RM. For each successful 3-RM trial the load was increased by a minimum of 2.5 kg until the subject was unable to reach full knee extension for three repetitions. A minimum of 2 min of rest was given between trials and 3-RM was generally determined within five trials. The subjects’ 1-RM was estimated from their 3-RM using the following equation; 1 RM = (3 RM)/[1.0278 − (3 × 0.0278)] (Brzycki, 1993).

### Muscle Biopsy Sampling and Preparation

Before and after the 6-week intervention period, muscle biopsies (~120 mg) were collected from *v. lateralis* distally to the occlusion site under sterile conditions and local anesthesia (1% Lidocaine, Mylan Hospital, Norway) using the Bergström needle technique (Bergstrom, 1975). All biopsies were harvested at ~1 to 2 cm depth. Pre- and post-intervention biopsies were collected from the same leg (randomized for leg dominance) with ~3 cm between sampling sites (Vissing et al., 2005). Immediately following collection, biopsies were dissected free of visible fat and connective tissue. Tissue for fractional synthesis rate (FSR) analysis (~50 mg) and immunoblotting (~30 mg) were frozen in liquid nitrogen. Tissue for immunohistochemical analysis (~40 mg) was embedded in Tissue-Tek (Leica Biosystems, Nussloch, Germany) and frozen in isopentane pre-cooled in liquid nitrogen. Biopsies were stored at −80°C.

### Tissue Preparation for Measurement of Myofibrillar Protein FSR

Body water enrichment and tissue alanine enrichment were determined from plasma as previously described (Robinson et al., 2011; Drake et al., 2013; Scalzo et al., 2014; Konopka et al., 2017). Approximately 25–50 mg of skeletal muscle was homogenized in an isolation buffer containing 100 mM KCl, 40 mM Tris HCl, 10 mM Tris base, 5 mM MgCl_2_, 1 mM EDTA, and 1 mM ATP (pH 7.5), with phosphatase and protease inhibitors (HALT; ThermoScientific, Rockford, IL, USA) with a bead homogenizer (Next Advance, Inc., Averill Park, NY, USA). After homogenization, the samples were centrifuged at 800 *g* for 10 min at 4°C. The resulting pellet enriched with myofibrillar proteins was isolated and washed with 500 μl of 100% ethanol and rinsed with 500 μl of distilled water twice. The pellet was resuspended in 250 μl of 1 M NaOH and placed on a heat block for 15 min at 50°C shaking at 900 rpm. The myofibrillar protein enriched fraction was then incubated in 6 N HCl for 24 h at 120°C for protein hydrolysis. The hydrolysates were ion exchanged, dried in a vacuum, and resuspended in 1 ml of molecular biology grade H_2_O. Half of the suspended sample was derivatized by a 1-h incubation of 500 μl acetonitrile, 50 μl K_2_HPO_4_, pH 11, and 20 μl of pentafluorobenzyl bromide. Ethyl acetate was added and the organic layer was removed, dried under nitrogen gas, and reconstituted in 600 μl ethyl acetate for analysis on an Agilent 7890A GC coupled to an Agilent 5975C MS as previously described (Robinson et al., 2011; Scalzo et al., 2014; Konopka et al., 2017). The newly synthesized fraction (*f*) of myofibrillar proteins was calculated from the enrichment of alanine bound in muscle proteins over the entire labeling period, divided by the true precursor enrichment (*p*), using the average plasma D_2_O enrichment over the period of measurement with MIDA adjustment (Busch et al., 2006).

### RNA Extraction and Measurement of FSR

Approximately 15–25 mg of skeletal muscle was homogenized in 800 μl of Trizol (ThermoFisher, Rockford, IL, USA) using a bead blender. The homogenate was centrifuged at 12,000 *g* for 10 min at 4°C. The resulting supernatant was removed and 160 μl of chloroform was added. The mixture was shaken vigorously then centrifuged at 12,000 *g* for 15 min at 4°C. The upper aqueous layer was isolated, mixed with 400 μl of isopropanol, and then left to incubate at room temperature for 10 min. After incubation, the mixture was centrifuged for 10 min at 4°C to pellet RNA. The RNA pellet was isolated, rinsed with 800 μl of 75% ethanol, and resuspended in 50 μl of molecular biology grade H_2_O. RNA synthesis (~85% of total RNA exists as ribosomal RNA) was determined by deuterium incorporation into purine ribose of RNA as previously published (Mathis et al., 2017). The isolated RNA was hydrolyzed overnight at 37°C with nuclease S1 and potato acid phosphatase. Hydrolysates were reacted with pentafluorobenzyl hydroxylamine and acetic acid and then acetylated with acetic anhydride and 1-methylimidazole. Dichloromethane extracts were dried, resuspended in ethyl acetate, and analyzed on an Agilent 7890A GC coupled to an Agilent 5975C MS. For GC-MS analysis, we used a DB-17 column and negative chemical ionization, with helium as carrier and methane as the reagent gas. The fractional molar isotope abundances at m/z 212 (M0) and 213 (M1) of the pentafluorobenzyl triacetyl derivative of purine ribose were quantified using ChemStation software. All analyses were corrected for abundance with an unenriched pentafluorobenzyl triacetyl purine ribose derivative standard. For the precursor enrichment, the average D_2_O enrichment over the period of measurement was adjusted by MIDA for ribose equilibration (Busch et al., 2006).

### Immunohistochemistry

Serial transverse 10 μm cross-sections were cut from the embedded biopsy at −18°C using a cryostat (CM3050S, Leica Biosystems, Nussloch, Germany) and mounted on glass slides (Superfrost Ultra Plus, Thermo Scientific, Germany). Cross-sections were stored at −80°C until later analysis.

#### Muscle Fiber Cross-Sectional Area

Muscle biopsy cross-sections were placed in room temperature and allowed to thaw and dry. Sections were fixed in Histofix (Histolab, Gothenburg, Sweden) for 4 min followed by 1.5 h blocking in blocking buffer (2% BSA, 5% FBS, 2% goat serum, 0.2% Triton x-100, 0.1% sodium azide). Sections were incubated overnight at 4°C in primary antibody MHC-I (1:1,000; cat. no. A4.951, Developmental Studies Hybridoma Bank, IA, USA) for distinction of muscle fiber type I. Next, sections were incubated with Alexa-fluor 568 goat anti-mouse (1:500; cat. no A11004, Molecular Probes, Invitrogen A/S, Taastrup, Denmark) secondary antibody for 1 h followed by incubation with 488 mouse anti-Human Collagen IV (1:100; cat. no. 53-9871, Affymetrix, CA, USA) antibody for 1 h for visualization of muscle fiber border. A cover slip was applied on sections using mounting medium (Cat. no. P36930, Molecular Probes Prolong Gold anti-fade reagent, Invitrogen A/S, Taastrup, Denmark) and stored at −20°C until further analysis. Washing in three changes of 1% PBS was carried out between all steps. Antibodies were diluted in 1% BSA.

Images were captured at 10× magnification with a Leica DM2000 microscope and a Leica DFC450 Hi-resolution Color DFC camera (Leica Microsystems, Broenshoej, Denmark). Muscle fiber border and muscle fiber type were identified using semi-automatic segmentation software (Smith and Barton, 2014). Manual correction of the initial segmentation and fiber-typing was made before determination of fiber-type-specific CSA and fiber-type distribution. Mean [95% CI] number of fibers included in the analysis of muscle fiber CSA and fiber-type distribution were 215 [188, 242] for type I fibers and 255 [217, 292] for type II fibers. Total fiber area was computed as a weighted mean of type I and type II CSA.

#### Satellite Cells and Myonuclei

Cross-sections were initially prepared as described above. After blocking, the sections were incubated overnight at 4°C in primary antibody Pax7 (1:500; cat. no, MO15020, Neuromics, MN, USA) for visualization of SCs followed by incubation in Alexa flour 568 goat-anti-mouse secondary antibody (1:200; cat. no. A11004, Invitrogen A/S, Taastrup, Denmark) for 1.5 h. Next, sections were incubated with a mixture of primary antibodies against MHC-I (1:500; cat. no. A4.951, Developmental Studies Hybridoma Bank, IA, USA) and laminin (1:500; cat. no. Z0097, Dako Norden, Glostrup, Denmark) for 2 h for distinction of muscle fiber type I and muscle fiber border, respectively. Secondary antibodies Alexa Fluor 488 goat anti-mouse and Alexa Fluor 488 goat anti-rabbit (1:500; cat. no A11029 and A11008, Invitrogen A/S, Taastrup, Denmark) were mixed an applied to the sections for 1 h. A cover slip was applied on sections using mounting medium containing 4′,6′-diamidino-2-phenylindole (DAPI) which stains the nuclei (Cat. no. P36935, Molecular Probes Prolong Gold anti-fade reagent, Invitrogen A/S, Taastrup, Denmark). Sections were stored at −20°C until further analysis. Washing in three changes of 1% PBS was carried out between all steps. Antibodies were diluted in 1% BSA.

Images were captured at 20× magnification with a Leica DM2000 microscope and a Leica DFC450 Hi-resolution Color DFC camera. The number of SCs associated with type I or type II fibers were quantified separately by counting cells characterized by co-localization of Pax7 and DAPI inside the basal lamina of distinct muscle fibers. Satellite cells (SC) were expressed relative to the total number of type I and type II fibers (SC/fiber) and CSA (SC/mm^2^). For quantification of myonuclei, we counted Pax7 negative nuclei with a geometric center within the basal lamina (Bruusgaard et al., 2012). Number of myonuclei was expressed relative to the total number of type I and type II fibers (myonuclei/fiber) and as myonuclear domain (μm^2^/myonuclei). The number of fibers analyzed for SCs and myonuclei was based on Mackey et al. (2009). For SC analysis, we counted a mean [95% CI] number of 154 [136, 172] type I fibers and 165 [144, 187] type II fibers. For myonuclei analysis, we counted 83 [77, 88] type I fibers and 89 [80, 97] type II fibers.

Immunohistochemical image analysis was conducted with the investigator blinded for subject ID and time of sample collection. Fibers situated on the edge of the cross-sections as well as fibers characterized by poor morphological integrity were excluded from analysis.

### Immunoblotting

Frozen muscle tissue was freeze-dried, homogenized, separated by sodium dodecyl sulfate poly-acrylamide gel electrophoresis (SDS-PAGE), and electroblotted onto PVDF membranes as previously described (Rahbek et al., 2015). Primary antibodies were purchased from Cell Signaling Technology (Danvers, MA, USA) and used as follows; p-FoxO3a (Ser^253^) (conc. 1:1000, cat # 3938) p-ULK1Ser555 (1:2,000; cat. no. 5869) and LC3B (1:1,000; cat. no. 3868). P-FoxO3a was diluted in 1% BSA. The other primary antibodies were diluted in 5% BSA. After overnight incubation in primary antibodies, membranes were incubated for 1 h with horseradish peroxidase-conjugated goat anti-rabbit (cat. no. 6721 ABCAM, Cambridge, UK) in a 1:5,000 solution with 1% BSA. Proteins were visualized by chemiluminescence (Thermo Scientific, Waltham, MA, USA) and quantified with an UVP imaging system (UVP, Upland, CA, USA). Arbitrary protein intensity was normalized to total amount of protein loaded in the corresponding lanes using Stain Free Technology as previously described (Gilda and Gomes, 2013; Gurtler et al., 2013).

### RNA Purification and Analysis

Approximately 15–30 mg of muscle tissue was homogenized using a Precellys 24 (Bertin Technologies, France). Purification of total RNA was carried out using a QIAGEN RNeasy Mini Kit (cat. #217004, QIAGEN, Germany) according to the manufacturer’s instructions. Quantification of RNA was determined by measuring the absorbance at 260 nm using a spectrophotometer (NanoDrop 1,000; Thermo Scientific, IL, USA). As ~85% of RNA exists as ribosomal RNA, total RNA (ng/mg tissue) was considered a marker of ribosomal abundance.

### Statistical Analysis

Differences between groups in myofibrillar MPS and RNA synthesis were evaluated with a one-way ANOVA. Statistical analysis of muscle fiber CSA, satellite cell, myonuclei, muscle strength, and immunoblotting data were performed using a linear mixed model with group, time, and time × group interaction as the factors of interest. Model validation included test for equal standard deviations and examination of QQ plots. Associations between variables were evaluated using linear regression and Pearson’s correlation. Using K-means cluster analysis, subjects in the two training groups were pooled and separated into non-responders and responders based on the magnitude of relative changes in total muscle fiber CSA as previously done by others (Stec et al., 2016). The cluster algorithm made the two clusters to minimize the sum of the squared distances to the cluster centers. Subsequently, analysis of differences between non-responders and responders were performed using a linear mixed model with cluster, time, and time × cluster interaction as the factors of interest. Alpha level was set to *p* ≤ 0.05. Graphic data are presented as mean ± SD. Data in tables and text are presented as mean with 95% CI. Statistical analysis was made in Stata 15.0 (Statacorp, College Station, TX, USA) and graphical presentations were made in GraphPad Prism version 7.0 (GraphPad Software, La Jolla, CA, USA).

## Results

### Subjects

All subjects completed the intervention. Baseline characteristics are presented in Table 1 in Groennebaek et al. (2018). Two subjects in the HLRE group completed 15 and 17 of the 18 scheduled training sessions. The remaining 20 subjects in the two training groups completed all 18 training sessions.

### Training Parameters

Training parameters averaged over the training period are presented in (Groennebaek et al., 2018). When compared to HLRE, BFRRE training was characterized by a higher number of performed repetitions, but a lower training load and training volume.

### Muscle Fiber-Type-Specific Cross-Sectional Area

**Figure 2:**
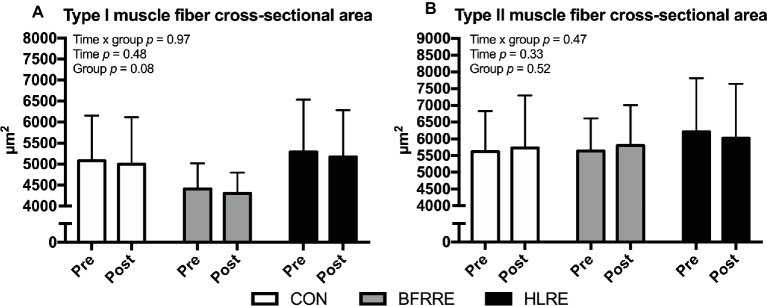
Muscle fiber cross-sectional area of type I fibers **(A)** and type II fibers **(B)** at baseline (pre) and 4 days after cessation of training (post). Overall effects are given in the upper left corner of graphs of **(A,B)**.

The effect of the intervention on changes in muscle fiber CSA is described using fiber-type-specific mean CSA (Figure 2) and area-frequency distribution (Supplementary Figure S1). As shown in Figure 2, no changes in the CSA of type I or type II fibers were observed in either group. Moreover, area-frequency curves revealed no changes in the proportion of smaller or larger fibers in any of the groups (Supplementary Figure S1).

### Long-Term Myofibrillar Protein Synthesis and RNA Synthesis

**Figure 3:**
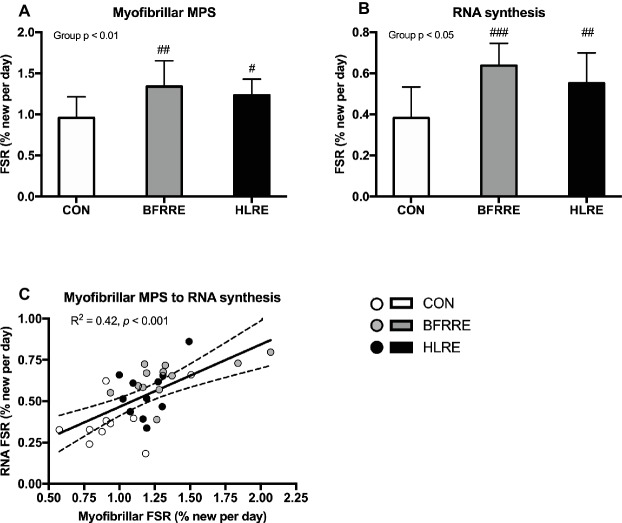
Myofibrillar protein **(A)** and RNA **(B)** synthesis rates (%/day) during the intervention period. Correlation between myofibrillar FSR and RNA FSR **(C)**. Data are presented as mean ± SD in **(A,B)**. In **(C)**, data are presented as individual values and a linear regression line (solid) with 95% CI (dashed). Overall group effect is given in the upper left corner of graphs of **(A)** and **(B)**. *R* square and significance is given in the upper left corner of **(C)**. ^#^*p* < 0.05, ^##^*p* < 0.01, and ^###^*p* < 0.001 different from CON.

Average D_2_O body water enrichment was stable throughout the labeling period as reported previously (Groennebaek et al., 2018). As shown in Figure 3, both training groups had higher myofibrillar MPS and RNA synthesis compared to CON. No differences in myofibrillar MPS or RNA synthesis were observed between BFRRE and HLRE. Myofibrillar protein FSR was correlated to RNA FSR (*R*^2^ = 0.42, *p* < 0.001; Figure 3A). However, neither RNA FSR nor myofibrillar protein FSR were correlated to the percentage change in total muscle fiber area (*R*^2^ = 0.09, *p* = 0.18, and *R*^2^ = 0.09, *p* = 0.16, respectively, data not shown).

### Total RNA

**Figure 4:**
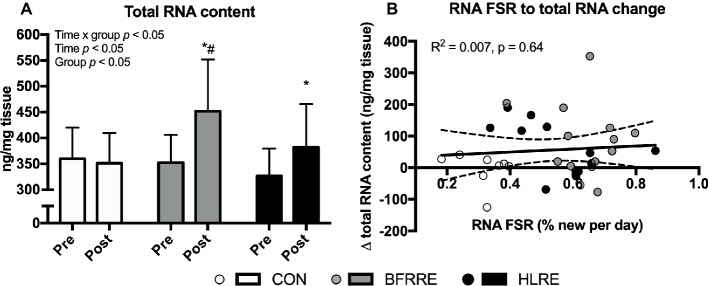
Total RNA content at baseline (pre) and 4 days after cessation of training (post) **(A)**. Correlation between RNA synthesis and change in total RNA content. Data are presented as mean ± SD in **(A)**. In **(B)**, data are presented as individual values and a linear regression line (solid) with 95% CI (dashed). Overall effects are given in the upper left corner of graphs **(A)**. R square and significance is given in the upper left corner of **(B)**. ^*^p < 0.05 different from pre within group; ^#^*p* < 0.01 different from CON.

Overall effects of time, group and time × group was detected for total RNA content (*p* < 0.05). As shown in Figure 4A, total RNA content increased from pre to post in BFRRE and HLRE. At post, total RNA content was higher in BFRRE compared to CON. No changes were observed in CON. No correlation between RNA synthesis and change in total RNA content was observed (Figure 4B). The change in total RNA content was not correlated to RNA synthesis (Figure 4B) or MPS (*R*^2^ = 0.004, *p* = 0.74, data not shown).

### Autophagy Signaling

**Figure 5:**
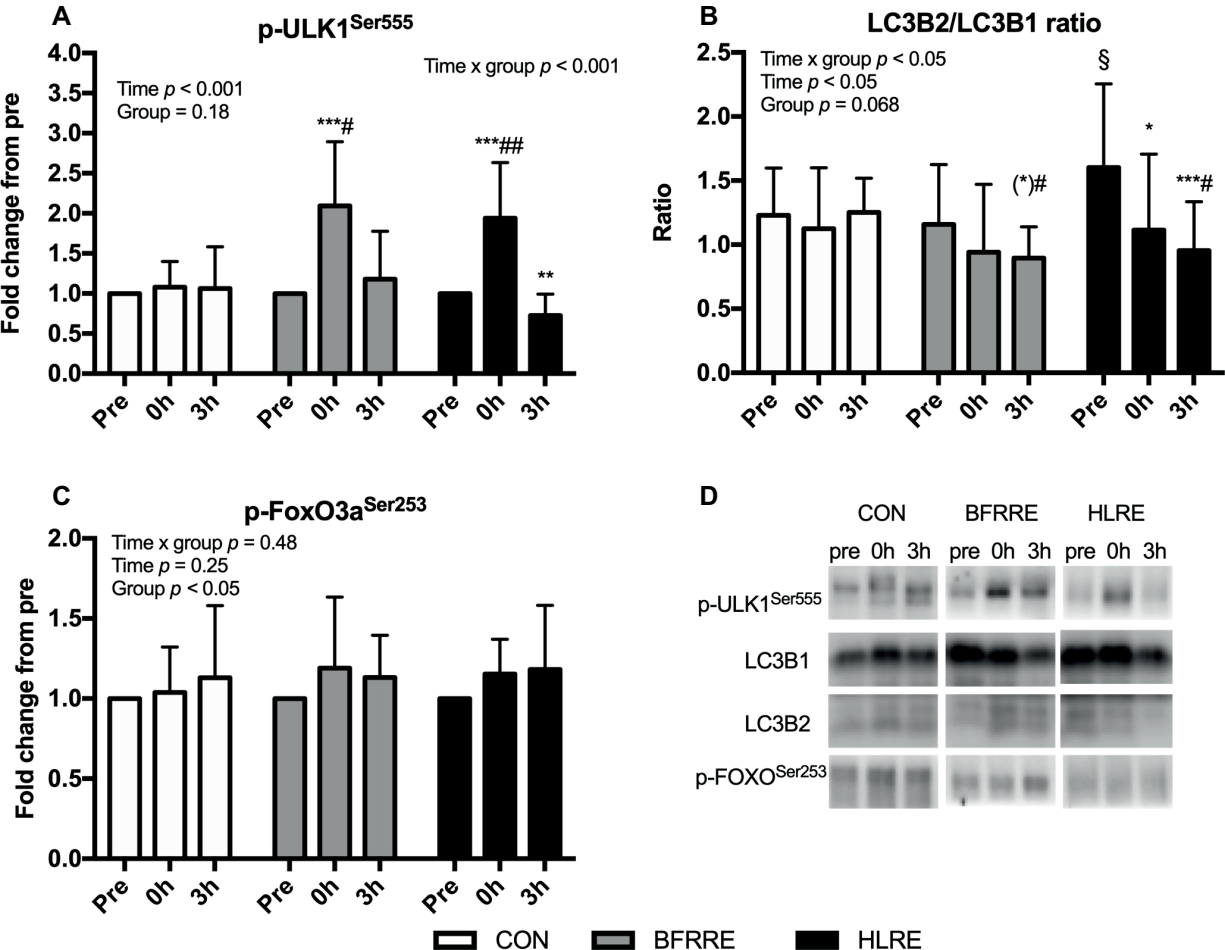
Phosphorylation of ULK1 at Ser^555^ **(A)**, the ratio of LC3B2 to LC3B1 **(B)** protein expression, and **(C)** phosphorylation of FoxO3a at Ser^253^ immediately (0 h) and 3 h (3 h) after acute exercise. Data are presented as mean ± SD. Overall effects are given in the upper left corner of graphs. ^*^p < 0.05, ^**^p < 0.01, and ^***^p < 0.001 different from pre within group; (*)*p* < 0.1 tendency toward difference from pre within group; ^§^*p* < 0.05 different from BFRRE within time-point; ^#^*p* < 0.05 and ^##^*p* < 0.01 different from CON within time-point. Representative blots are shown in **(D)**.

BFRRE and HLRE increased phosphorylation of ULK1 at Ser^555^ immediately (0 h) following exercise (Figure 5A). Moreover, the ratio of LC3B2 to LC3B1 decreased after HLRE and tended to decrease 3 h after BFRRE (Figure 5B). In HLRE, this was mainly driven by a decreased protein expression of LC3B2 while the tendency in BFRRE emerged owing to non-significant up- and downregulation of LC3B1 and LC3B2 expression, respectively (data not shown). No changes in phosphorylation of FoxO3a at Ser^253^ were observed in any of the groups (Figure 5C). No changes were observed in CON in any of the autophagy-related targets analyzed. Representative immunoblots are shown in Figure 5D.

### Satellite Cells

**Figure 6:**
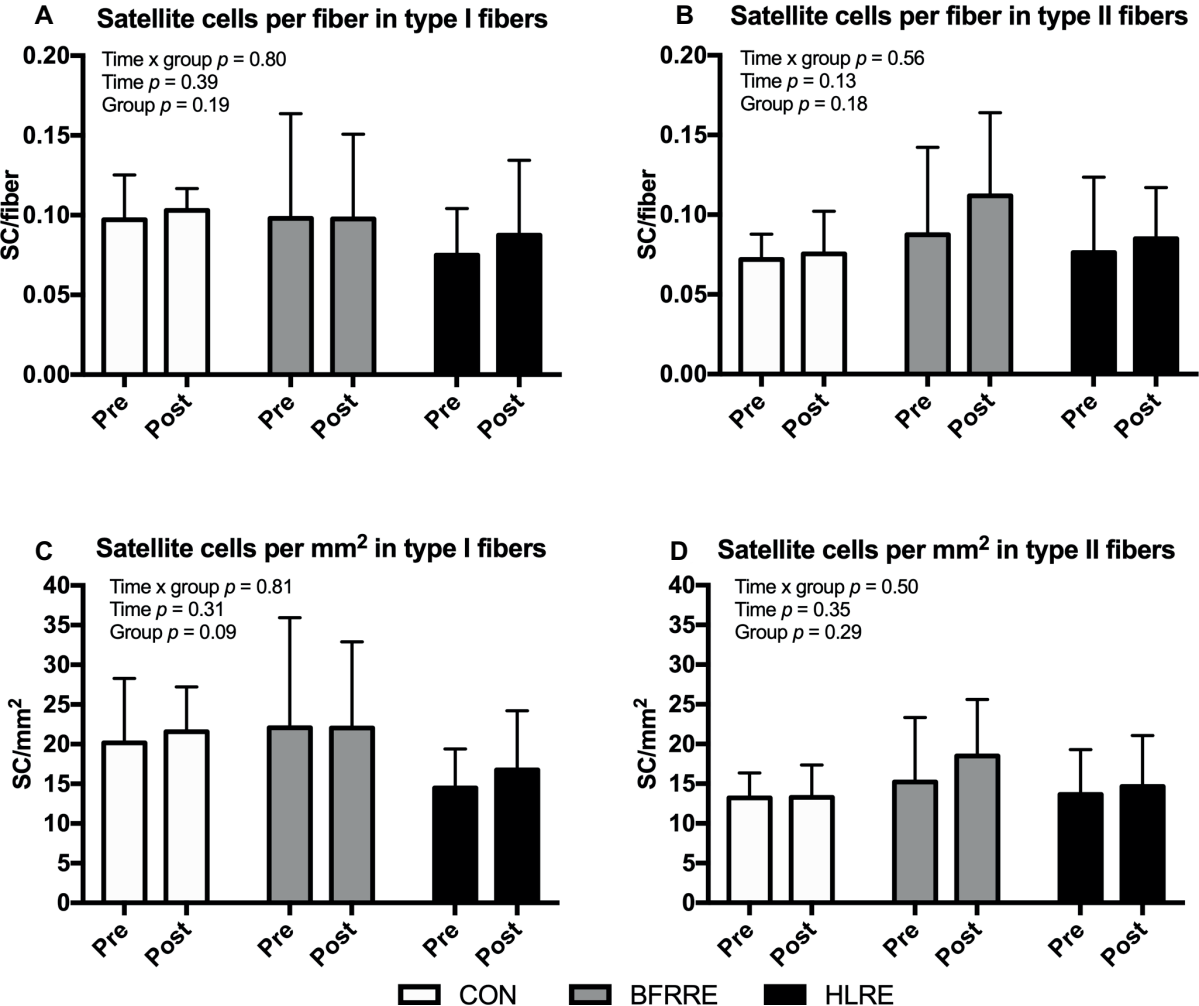
Number of satellite cells at baseline (pre) and 4 days after cessation of training (post) expressed relative to number of fibers **(A,B)** and fiber CSA **(C,D)**. Data are presented as mean ± SD. Overall effects are given in the upper left corner of graphs.

No changes in fiber-type-specific number of satellite cells were observed when satellite cells were normalized to number of fibers (Figures 6A,B) or fiber CSA (Figures 6C,D). Similarly, no changes were observed when the number of satellite cells were expressed as a percentage of the total number of nuclei [SC/(SC + myonuclei) × 100] (Supplementary Figure S2).

### Myonuclei

**Figure 7:**
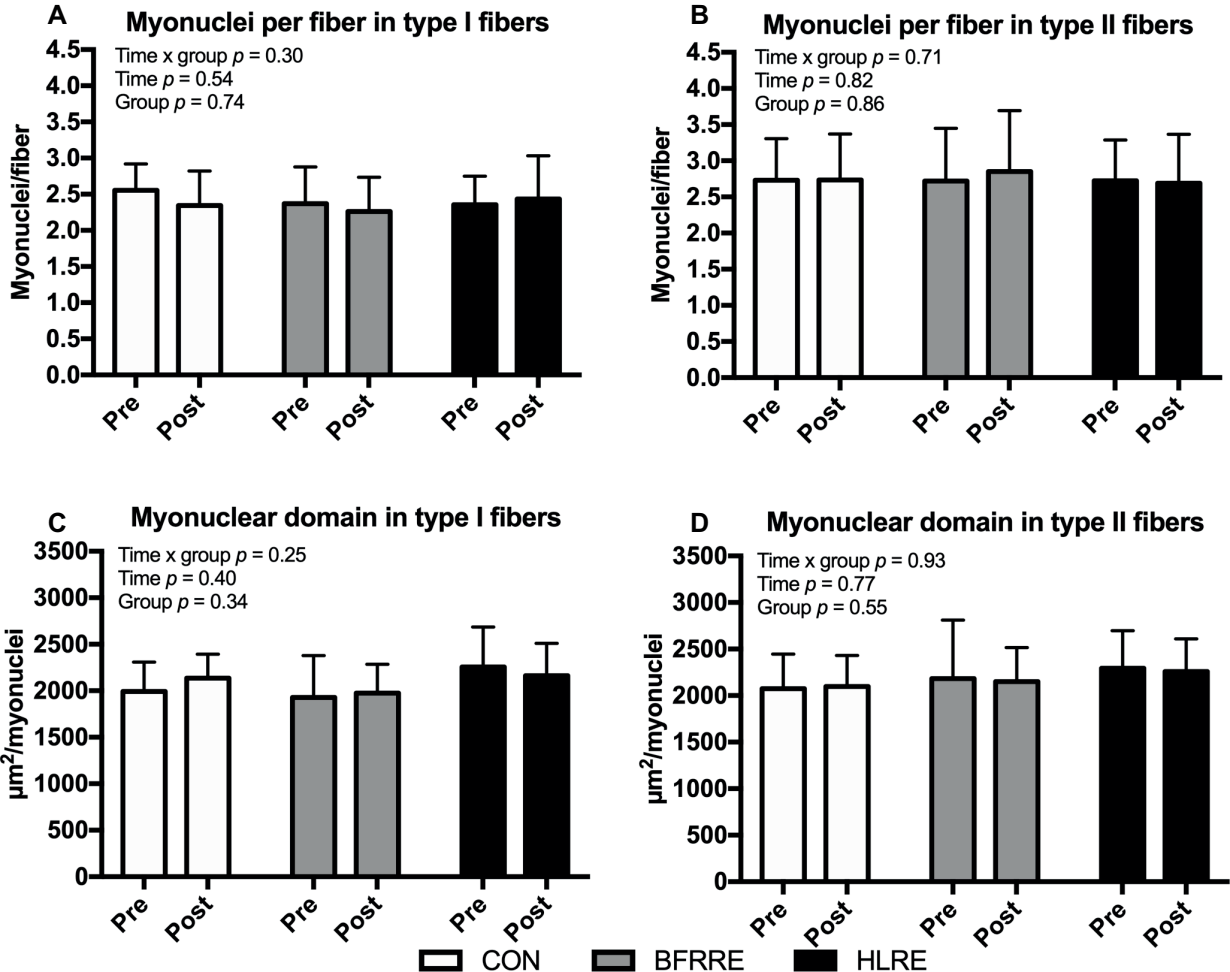
Myonuclei at baseline (pre) and 4 days after cessation of training (post) expressed relative to number of fibers **(A,B)** and as myonuclear domain **(C,D)**. Data are presented as mean ± SD. Overall effects are given in the upper left corner of graphs.

As shown in Figure 7, no changes in fiber-type-specific number of myonuclei per fiber or myonuclear domain were observed.

### Muscle Strength

**Figure 8:**
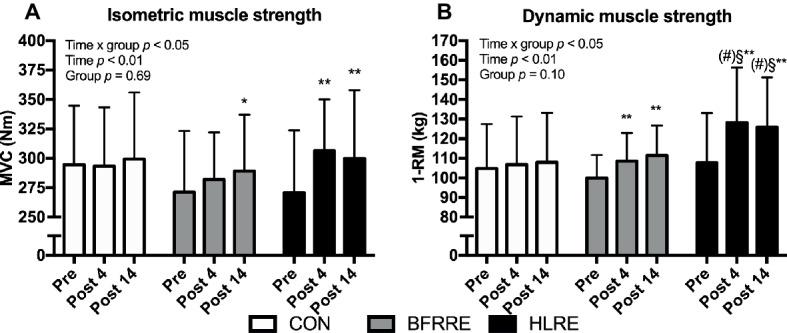
Isometric muscle strength **(A)** and dynamic muscle strength **(B)** at baseline (pre), 4 days after cessation of training (post 4), and 14 days after cessation of training (post 14). Data are presented as mean ± SD. Overall effects are given in the upper left corner of graphs. ^*^p < 0.05 and ^**^p < 0.01 different from pre within group; ^§^*p* < 0.05 different from BFRRE within time-point; (#) *p* < 0.1 tendency toward difference to CON within time-point.

Changes in muscle strength are shown in Figure 8.

#### Isometric Muscle Strength (MVC)

An overall time × group interaction for MVC was detected (*p* < 0.05). No changes were observed in CON. HLRE increased MVC from pre to post (13.6 [4.8, 22.4]%) and from pre to 14 days after cessation of training (13.1 [3.4, 22.8]%). In BFRRE, MVC increased from pre to 14 days after cessation of training (6.2 [−0.3, 12.6]%). No between-group differences were observed.

#### Dynamic Muscle Strength (1-RM)

An overall time × group interaction for 1-RM was detected (*p* < 0.05). No changes were observed in CON. Both training groups increased 1-RM from pre to post (BFRRE 8.7 [3.8, 13.6]%; HLRE 19.6 [11.8. 27.4]%) and from pre to 14 days after cessation of training (BFRRE 9.90 [3.5, 16.3]%; HLRE 22.5 [14.1, 30.9]). Differences between BFRRE and HLRE were observed at post 4 and post 14. A tendency toward a difference between HLRE and CON was observed at post 4 (*p* = 0.067) and post 14 (*p* = 0.072).

### Characteristics of Non-Responders and Responders

**Figure 9:**
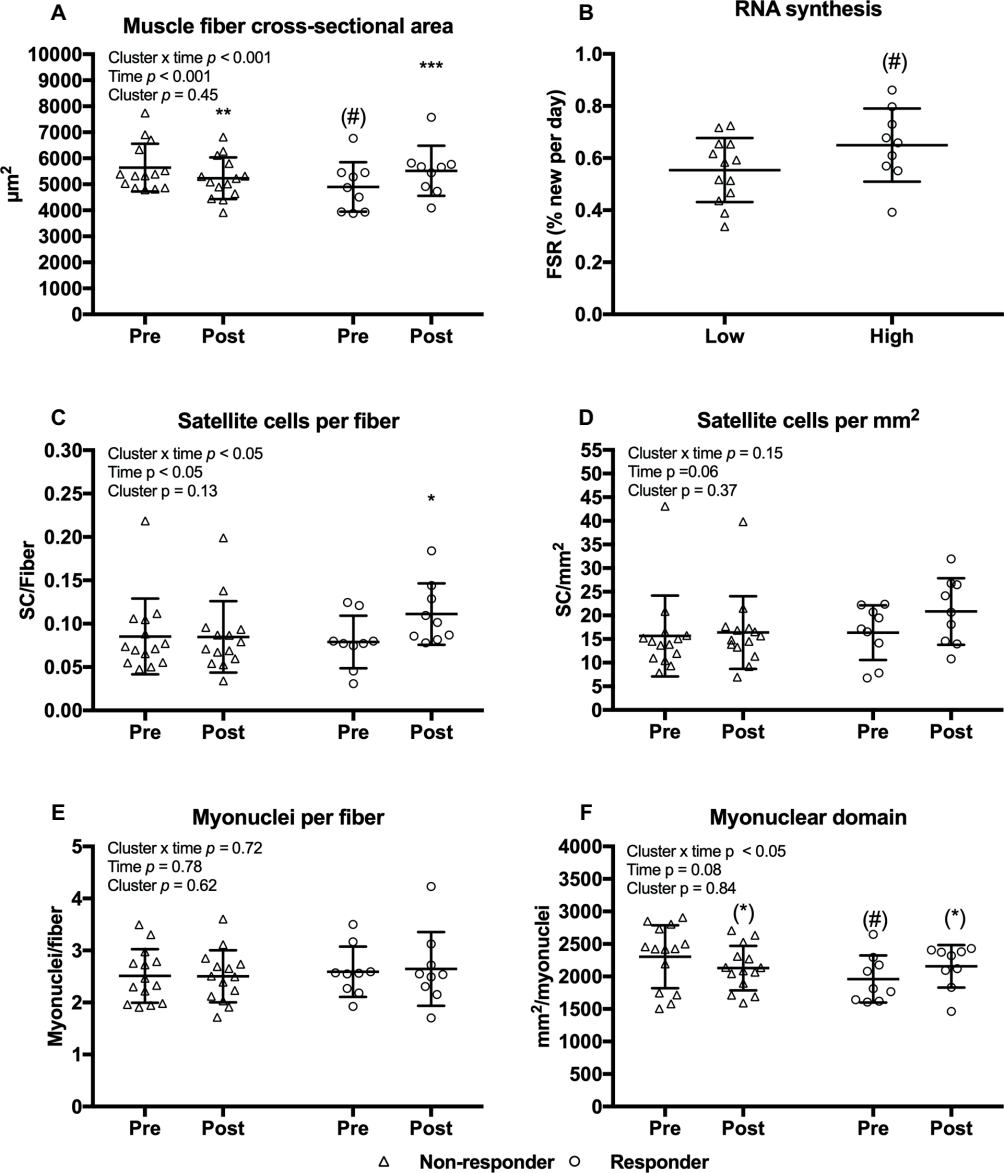
Muscle fiber cross-sectional area **(A)**, satellite cells per fiber **(C)**, satellite cells per mm^2^ **(D)**, myonuclei per fiber **(E)**, and myonuclear domain **(F)** at baseline (pre) and 4 days after cessation of training (post) in non-responders (*n* = 14) and responders (*n* = 9) with regards to muscle fiber hypertrophy. RNA synthesis in non-responders and responders **(B)**. Data are presented as individual values as well as mean ± SD. Overall effects are given in the upper left corner of graphs. (*)*p* < 0.1 tendency toward difference from pre within cluster. ^*^p < 0.05, ^**^p < 0.01, and ^***^p < 0.001 different from pre within cluster; (#)*p* < 0.1 tendency toward difference to non-responders within time-point.

Given a large variability in the changes in muscle fiber CSA, and the additional insight gained by examining variable responses (Stec et al., 2016) we performed an analysis of responders versus non-responders to the current training regimen. Clustering of non-responders and responders were based on the magnitude of relative changes in total muscle fiber CSA (weighted mean of type I and II CSA). Mean changes in fiber CSA were −7.0 [−11.1, −2.89]% in non-responders and 13.3 [6.8, 19.8]% in responders (Figure 9A). A tendency toward lower fiber CSA and myonuclear domain (Figures 9D,E) were observed before the training intervention in the non-responders compared to responders (Figures 9A,F). Training led to an increase in number satellite cells per fiber in responders only (Figure 9C). Furthermore, RNA synthesis tended to be higher in responders compared to non-responders (Figure 9B). No differences between clusters were observed in myofibrillar MPS (*p* = 0.36) or total RNA content (*p* = 0.92) (data not shown).

## Discussion

The present study comprehensively investigated skeletal muscle adaptive responses to 6 weeks of BFRRE or HLRE conducted by recommended exercise principles. The main findings were; (1) that BFRRE and HLRE produced similar increases in long-term myofibrillar MPS and RNA synthesis without concomitant increases in muscle fiber CSA; (2) that increases in satellite cell content was observed in responders, and; (3) that muscle strength increased with both training regimens, albeit to greater extent with HLRE. The present data support the potential of low-load BFRRE as an alternative training modality in clinical settings.

With regard to the training regimens, we deliberately chose a rather short training period. In accordance, the effectiveness of a ≤6-week resistance training period has previously been demonstrated as capable of producing muscle hypertrophy (Nielsen et al., 2012; Fahs et al., 2015; Holloway et al., 2018; Bjornsen et al., 2018a). Moreover, we utilized recommended exercise principles (i.e., BFRRE; low load, many repetitions, short inter-set recovery and HLRE; high load, few repetitions, long inter-set recovery; ACSM, 2009; Scott et al., 2015). This approach was chosen to resemble how exercise is practiced outside the laboratory rather than attempting to appoint one of the several differences between BFRRE and HLRE (i.e., ischemia, load, volume, and inter-set recovery) as more important for standardization.

### Effects of BFRRE and HLRE on Myofibrillar Protein and RNA Synthesis

Previous studies utilizing short-term primed continuous amino acid infusions have reported that low-load BFRRE as well as HLRE can stimulate acute increases in myofibrillar MPS (Phillips et al., 1997; Fujita et al., 2007; Fry et al., 2010). A study by Burd et al. (2010) reported that mixed MPS was augmented at 24 h after single-bout low-load compared to high-load resistance exercise, but this study did not employ blood flow restriction. Notably, the MPS response to an acute bout of exercise can persist for ~24–72 h post-exercise (Phillips et al., 1997; Miller et al., 2005; Damas et al., 2016). The current study employed an approach using D_2_O to assess cumulative myofibrillar MPS. This approach allows for assessment of long-term protein synthesis under free-living conditions and better accounts for proteins with slower turnover rates and/or less abundance (Miller et al., 2015). Using this approach, we found an increase in cumulative myofibrillar MPS after HLRE, which is in accordance with recent studies using D_2_O (Brook et al., 2015, 2016). Interestingly, low-load BFRRE produced a similar increase in myofibrillar MPS as HLRE. These findings support the notion that BFRRE provides a low-load approach to stimulate long-term protein turnover. This notion is further supported by our novel findings of increased cumulative RNA synthesis and increased total RNA content with BFRRE as well as HLRE. Since ~85% of total RNA exists as ribosomal RNA (Zak et al., 1967), increases in cumulative RNA synthesis and content observed by ourselves and others (Figueiredo et al., 2015; Brook et al., 2017) therefore primarily likely reflects increased ribosomal biogenesis and content, which may yield an increased translational capacity. In the current study, changes in RNA content were not correlated to RNA synthesis. This would suggest that rates of ribosomal biogenesis cannot quantitatively predict increases in ribosomal content and changes in ribosomal content is vice versa not indicate of how much new is made. As with protein content, ribosomal content is regulated by synthesis and breakdown. Ribosome breakdown was not assessed here, but we speculate that the observed increase in cumulative RNA synthesis was likely targeted for both ribosome accretion and ribosome maintenance/repair.

In both the current study, and the study by Brook et al. (2017), RNA synthesis and MPS were strongly correlated. This is further corroborated by studies reporting that *in vitro* protein synthesis is highly dependent on ribosomal content and biogenesis (Stec et al., 2016; West et al., 2016). Interestingly, RNA synthesis, but not RNA content, was correlated to MPS in our study which would suggest that the ability to increase MPS *in vivo* may preferentially be tied to the ability to make new ribosomes, rather than to increase the overall ribosome content On the other hand, previous human resistance training studies have advocated that changes in RNA content are in fact correlated to muscle hypertrophy (Figueiredo et al., 2015), which would naturally be preceded by increases in MPS. Thus, available evidence from our study and previous investigations strongly indicate that ribosomal biogenesis and MPS are involved in a coordinated regulation of protein turnover in response to exercise. However, we acknowledge that the utilization of a 6-week measurement period does not allow us to decipher whether the synthetic responses were primarily driven by large increases in the early phase of the training period, so this aspect warrants further investigation.

The heterogeneity of response between individuals to resistance exercise training is increasingly appreciated (Stec et al., 2016). Knowledge on the heterogeneity is important for appropriate exercise prescription as well as determining mechanisms that dictate muscle growth. A previous study from Bamman and co-workers stratified responders and non-responders to a resistance training protocol and demonstrated that an increase in ribosomal content was a key differentiating factor between the groups (Stec et al., 2016). In the current study, we did not observe a correlation between neither RNA synthesis nor RNA content and changes in muscle fiber CSA (*R*^2^ = 0.09, *p* = 0.18; *R*^2^ = 0.000, *p* = 0.87). However, when we performed a cluster analysis by pooling the subjects and separating responders from non-responders according to their individual change in muscle fiber CSA, there was a tendency (*p* = 0.098) toward a higher rate of RNA synthesis in responders. We observed no difference in RNA content between clusters but HLRE studies with a larger sample size than the current study have found that increases in RNA content are significantly greater in responders (Stec et al., 2016; Mobley et al., 2018). Ribosomal biogenesis therefore seems to constitute one underlying factor determining the muscle hypertrophic response to exercise.

To assess whether resistance training led to an increase in transcriptional capacity from satellite cell-mediated addition of myonuclei to existing muscle fibers, we analyzed satellite cell and myonuclei content. No changes in satellite cell or myonuclei content were observed. Only responders (as clustered based on relative change in muscle fiber CSA) showed a modest increase in satellite cell content. This suggests that under the current conditions, the transcriptional capacity of existing myonuclei was generally sufficient to provide mRNA transcript material for increased MPS [for further insight on this, we refer to Figueiredo and McCarthy (2019)].

Unlike the lack of change in satellite cell content in the current study, one previous study by Nielsen et al. (2012) reported remarkable increases in satellite cell content after BFRRE training. The Nielsen study also observed large increases in muscle fiber CSA. However, it should be noted that this study employed 23 exercise sessions in 19 days. In contrast, we employed 18 sessions over the course of 6 weeks, which obviously allowed for much more extended recovery between exercise sessions. The influence of recovery between BFRRE exercise sessions on satellite cells and fiber growth deserves further attention.

### Increased Protein Turnover With BFRRE and HLRE May Reflect Muscle Remodeling

Based on previous D_2_O studies showing correlations between cumulative increases in MPS and muscle hypertrophy (Brook et al., 2015; Damas et al., 2016), we hypothesized that an increase in MPS would lead to detectable muscle accretion as reflected by an increase in muscle fiber CSA. However, fiber-type-specific CSA as well as area-frequency distribution remained unchanged in all groups. This is in contrast to previous reports of increased muscle fiber CSA following BFRRE and HLRE training of comparable or even shorter duration than in our study (Staron et al., 1991; Goreham et al., 1999; Nielsen et al., 2012; Holloway et al., 2018; Bjornsen et al., 2018a,b). Nonetheless, it is debatable whether the low-volume single-exercise type of training, as employed in the current study, is sufficient to promote detectable muscle fiber hypertrophy after 6 weeks (McGlory et al., 2017) when also considering the large variation inherent of the single-biopsy technique (Lexell et al., 1985; Lexell and Taylor, 1989). Independent studies that have used ultrasound and magnetic resonance imaging (MRI) techniques, which have a generally high reliability in measuring whole muscle CSA (Franchi et al., 2018), have reported detectable hypertrophy following BFRRE as well as HLRE training (Kacin and Strazar, 2011; Ellefsen et al., 2015; Sieljacks et al., 2018) of shorter or comparable length than ours. For instance, using MRI we recently found CSA increases of ~8% in VL and ~3% in *m. quadriceps* following a comparable BFRRE training intervention in a similar population (Sieljacks et al., 2018).

The finding that 6 weeks of BFRRE and HLRE training increased MPS without any changes in muscle fiber CSA suggests that BFRRE and HLRE may have stimulated muscle remodeling. We were not able to assess bulk changes in muscle protein breakdown with use kinetic stable isotope methodologies. To provide indicatory information on activation on protein degradation, we analyzed biomarkers of protein degradation signaling. FoxO3 signaling is involved in both proteasomal and autophagy-related gene expression (Zhao et al., 2007; Milan et al., 2015) for E3 ligase gene transcription inherent of the ubiquitin proteasome system which is activated during atrophy conditions. We did not observe changes in FoxO3 phosphorylation. Autophagy constitute a catabolic process known to enable cellular remodeling by delivering dysfunctional proteins and organelles to the lysosomes for degradation (Boya et al., 2013; Bell et al., 2016). In previous studies, it was shown that an acute bout of exercise stimulates autophagy signaling through ULK1 in human skeletal muscle (Moller et al., 2015, 2018). Animal studies support that exercise stimulate increases in autophagy flux and that this activation is necessary to attain training adaptations. Consequently, our data support that autophagy signaling through ULK1 is stimulated during an acute bout of BFRRE and HLRE to engage in remodeling processes. However, it needs to be emphasized that our data constitute merely markers of degradation as no method exist to assess autophagy flux in human tissues *in vivo*. Moreover, we acknowledge that other biomarkers of protein degradation pathways (such as calpain and ubiquitin proteasomal systems) and/or other time points of measurement must be included in future studies, to provide for strengthened conclusions. Finally, it should be acknowledged that subjects were not accustomed to the exercise stimuli prior to the single-bout trial, which may likely affect signaling outcome (Wilkinson et al., 2008). However, our results support the notion that BFRRE and HLRE, increased protein turnover by stimulation of muscle MPS and muscle protein degradation. The ability of BFRRE to presumably stimulate muscle protein turnover may emphasize the potential application of BFRRE in ageing and disease settings even though no hypertrophy was observed, as protein turnover is critical during ageing and disease to prevent accumulation of damaged proteins (Haus et al., 2007; Gouveia et al., 2017; Musci et al., 2018). Noteworthy, the current study was made on healthy subjects and future studies should explore these responses in clinical populations.

### Effects of BFRRE and HLRE on Muscle Functional Capacity

HLRE produced an increase in MVC as well as maximal dynamic strength measured at both 4 and 14 days after cessation of training. Similar increases were produced with BFRRE. Yet, the increase in MVC with BFRRE did not reach statistical significance until 14 days after training. Similar observations of delayed maximal strength gains after BFRRE training have been reported previously (Nielsen et al., 2012), and it was to be owing to impaired intrinsic muscle function (Nielsen et al., 2017) Alternatively, the strength increase at post 14, could potentially relate to a learning effect between testing sessions.

The apparent greater ability of HLRE to enhance maximal strength has also been reported in other comparative studies (Karabulut et al., 2010; Yasuda et al., 2011; Martín-Hernández et al., 2013). The greater strength gains with HLRE may relate to differential neural innervation patterns/adaptations between BFRRE and HLRE. HLRE has been shown to induce greater EMG-assessed muscle activation during acute exercise compared to BFRRE (Cook et al., 2013), indicating greater motor unit recruitment and/or firing rates (Suzuki et al., 2002). Similarly, long-term HLRE has been shown to increase muscle activation following long-term training while no changes occurred following BFRRE (Kubo et al., 2006). It therefore seems that HLRE constitutes a stronger driver of maximal strength than BFRRE in young healthy subjects.

## Conclusion

The current study demonstrates that BFRRE and HLRE stimulate protein turnover, RNA synthesis, and increase muscle strength. These adaptations could be beneficial during ageing and disease to maintain protein homeostasis, muscle mass, and mobility. Owing to the low load, BFRRE may constitute a feasible and time-efficient training modality for certain clinical populations. Future studies should investigate the use of BFRRE in such populations.

## Ethics Statement

Written informed consent was obtained from all participants prior to inclusion. The study was approved by the Central Denmark Region Committee on Health Research Ethics (1-10-72-218-16) and registered in the database clinicaltrials.gov (NCT03380663). The study conformed to the standards for human experimental trials outlined in the Declaration of Helsinki.

## Author Contributions

The study was conducted at Section for Sports Science, Department of Public Health, Aarhus University. PS, TG, ER, FP, KH, BM, and KV contributed to conception and design. All authors contributed to data acquisition and/or interpretation of data. PS and KV wrote the first manuscript draft. All authors critically revised the manuscript and provided intellectual contributions. All authors approved the final version of the manuscript submitted for publication.

### Conflict of Interest Statement

The authors declare that the research was conducted in the absence of any commercial or financial relationships that could be construed as a potential conflict of interest.
