# Supplementary material for: Six Weeks of Low-Load Blood Flow Restricted and High-Load Resistance Exercise Training Produce Similar Increases in Cumulative Myofibrillar Protein Synthesis and Ribosomal Biogenesis in Healthy Males
Source: Front Physiol. 2019 May 29;10:649. doi: 10.3389/fphys.2019.00649 (PMC6548815; doi:10.3389/fphys.2019.00649)
Supplement: Supplementary file 1 [file Table_1.DOCX]

**Supplementary Material**

**Figure S1** Muscle fiber area-frequency distribution of type I and type II fibers presented as mean curves before and after 6 weeks of CON (A, B), BFRRE (C, D), or HLRE (E, F).

**Figure S2** Relative number of satellite cells expressed as a percentage of the total number of nuclei [SC/(SC + myonuclei) x 100] in type 1 (A) and type 2 (B) fibers. Data are presented as mean ± SD. Overall effects are given in the upper left corner of graphs.
